# Suppression of lncRNA *Gm47283* attenuates myocardial infarction via *miR-706*/ *Ptgs2*/ferroptosis axis

**DOI:** 10.1080/21655979.2022.2065743

**Published:** 2022-04-29

**Authors:** Feng Gao, Yongcheng Zhao, Bin Zhang, Chunwei Xiao, Zhanfa Sun, Yuan Gao, Xueyong Dou

**Affiliations:** Department Cardiovascular Surgery, Xuzhou Cancer Hospital, Xuzhou City, Jiangsu, China

**Keywords:** LncRNA *Gm47283*, myocardial infarction, ferroptosis, *Ptgs2*, *miR-706*

## Abstract

Myocardial infarction (MI) is the leading cause of sudden death. Long non-doing RNAs (lncRNAs) were demonstrated to play crucial roles in multiple diseases, including cancer and cardiovascular diseases. Nevertheless, the molecular mechanism of lncNRAs in MI is unclear. In this study, we integrated bioinformatics and molecular biological experiments to identify the novel lncRNA transcripts and elucidated its regulatory mechanism in MI. First, we identified 10 dysregualted lncRNAs and found that lncRNA *Gm47283* was the top risk factor in MI. Bioinformatics analysis predicted that lncRNA *Gm47283* exerted function via targeting *miR-706* and *Ptgs2. Ptgs2* was also the known regulator of ferroptosis. Inhibition or overexpression of lncRNA *Gm47283* could regulate *Ptgs2* expression and downstream ferroptosis activity. Overexpression of *miR-706* could inhibit the expression of *Ptgs2* and the activity of ferroptosis, thereby attenuated cellular injury. Mechanically, co-transfection experiments showed that overexpression of *miR-706* could reverse the damage effect that was caused by lncRNA *Gm47283* overexpression, via inhibiting *Ptgs2* and ferroptosis. Additionally, inhibition of lncRNA *Gm47283* by stem cell membrane coated siRNA could attenuate MI in *vivo*. Our study elucidated a novel mechanism containing lncRNA *Gm47283*/*miR-706*/*Ptgs2*/ferroptosis in MI, which provided a potential therapeutic for MI.

Graphical Abstract. Stem cell membrane coated siRNA of lncRNA *Gm47283* inhibits cardiomyocyte ferroptosis in myocardial infarction rat. Stem cell membrane-coated siRNA of lncRNA *Gm47283* increases *miR-706*, and then *miR-706* suppresses the *expression of Ptgs2* to reduce lipid peroxidation toxicity, and then inhibits cardiomyocyte ferroptosis. PUFA: polyunsaturated fatty acid.

## Graphical Abstract

**Figure:**
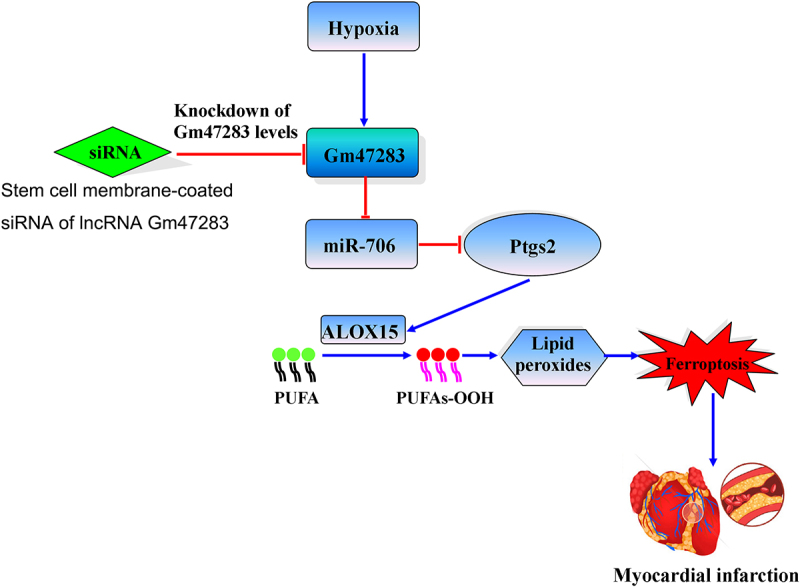

## Highlights

- It was confirmed that ferroptosis was related to myocardial infarction.
- We identified lncRNA Gm47283 was the top risk factor in myocardial infarction.
- We found that lncRNA Gm47283 exerted function via targeting miR-706 and Ptgs2 in myocardial infarction.
- For the first time, we used stem cell membrane coated siRNA of lncRNA Gm47283 to treat myocardial infarction.

## Introduction

Acute myocardial infarction (AMI) is mainly caused from coronary artery diseases, which is considered as the leading cause of morbidity and mortality worldwide [1]. Recent studies have focused on identifying the early diagnosis markers and discovering novel clinical treatments for patients of AMI to improve the survival levels [2]. However, the molecular mechanism of AMI is complex. Previous studies have found that some biological processes, such as apoptosis, fibrosis and inflammation are involved in the pathological processes of AMI [3–6]. Therefore, it is urgent to find the novel regulator and its regulatory axes of AMI and provide more information for clinical treatment.

Non-coding RNAs (ncRNAs), especially micro RNAs (miRNAs) and long non-coding RNAs (lncRNAs) have been reported to play key roles in the physiological and pathological processes of MI, such as *miR-21, miR-133,* and lncRNA *ZFAS1* [7–9]. Mechanically, miRNAs were demonstrated to exert function via targeting the 3’ untranslated region (3ʹUTR) of protein coding genes and gene body of lncRNAs [10]. Additionally, the newly proposed lncRNA is also demonstrated as the crucial regulators of complex pathological processes in cardiovascular diseases. Briefly, lncRNAs are defined as the transcript with more than 200 nucleotides and without non-protein coding ability, which involve in regulatory processes mainly via regulating gene transcription, translation, and alternative splicing in transcriptional and post-transcriptional levels [11]. Importantly, lncRNAs, miRNAs, and downstream protein coding genes can compose the close regulatory axes, which are named as competitive endogenous RNAs (ceRNAs). In the model of ceRNAs, activation of lncRNAs can protect the degradation of downstream protein coding genes via sponging common miRNAs. Studies have found that lncRNAs regulate MI through ceRNA regulatory mechanisms. For instance, Bin et al. have demonstrated that lncRNA *CRRL* suppresses cardiomyocyte regeneration via functioning as the endogenous decoy of *miR-199a-3p* and thereby activate the expression of *Hopx*, which leads to increased cardiac injury [12]. Wang et al. found that lncRNA *APF* can regulate *miR-188-3p*, and thus affects *ATG7* expression, autophagic cell death, and MI [13]. Hu et al. found that inhibition of *MALAT1* can attenuate myocardial apoptosis through suppressing *Pten* expression by sponging *miR-320* in AMI mouse [14]. All these studies inspire us to investigate the mechanism of AMI through lncRNA mediated ceRNAs perspective.

The loss of cardiomyocytes is the typical phenomenon during AMI, which is validated from numerous necrocytosis processes, like apoptosis, pyroptosis, autophagy, and ferroptosis [5,15–17]. Ferroptosis is a novel form of regulated cell death identified as iron-dependence, lipid peroxidation collection, and reactive oxygen species (ROS) generation [18–20]. It is remarkably distinct from other types of classical regulated cell death, such as apoptosis, necrosis, and necroptosis. Ferroptosis participated in various diseases, including cancer, Parkinson’s disease, stroke, and IRI [21–24]. However, its effect in the progression of AMI remains unclear.

In this study, we firstly used a bioinformatics pipeline to identify risk lncRNAs in MI based on microarray data. Results showed that 10 lncRNAs were dysregulated in MI. In *vivo* and in *vitro* models validated that lncRNA *Gm47283* was the high risk factor of MI. In addition, *TNF* and *NF-κB* signaling pathways were interpreted as activated regulatory processes in MI. And four genes were both enriched in the two pathways were annotated, including ferroptosis-related gene *Ptgs2*. LncRNA *Gm47283* and *Ptgs2* were also activated in MI models in *vivo* and in *vitro*. Bioinformatics analysis predicted that lncRNA *Gm47283* could regulate *Ptgs2* via endogenous sponging *miR-706*. Inhibition or overexpression of lncRNA *Gm47283* could regulate *Ptgs2* expression and ferroptosis activity. Mechanically, we found that overexpression of *miR-706* could attenuate the *Gm47283*-induced damage effects, via regulating cardiomyocyte ferroptosis activity by targeting *Ptgs2*. Moreover, inhibition of lncRNA *Gm47283* by nanometer materials coated siRNA could attenuate MI in *vivo*. Our study revealed a novel role for lncRNA *Gm47283* in regulating MI via ferroptosis pathway and lncRNA *Gm47283*/*miR-706*/*Ptgs2* regulatory axis could provide more information for therapeutic target design of MI.

## Methods

### Ethics statement

The procedures for the use of animals (c57bl/6 mouse; 20–25 g) in this work were in accordance with the regulations of the Ethic Committees of Xuzhou Cancer Hospital (ethics approval number: 2022–03-002-K01) and conformed to the National Research Council (NRC) Guide for the Care and Use of Laboratory Animals (2011, 8th edition).

### Bioinformatics analysis

We downloaded the raw expression data matrix and processed the gene expression of MI from GEO database with the accession number of GSE153485. We only reserved the samples of four groups: Sham_6 h group, Sham_24 h group, MI_6 h group and MI_24 h group (20 samples). We merged the groups into two groups, Sham group and MI group. Limma method was used to identify differentially expressed lncRNAs and genes with the threshold of 1.5 fold change or *P*-value <0.05. Protein–protein interaction network was extracted from STRING database by mapping the up-regulated genes [25]. Degree analysis of the network was performed via Cytoscape.

We used miRanda software to identify the downstream miRNAs of lncRNA *Gm47283*. Furthermore, we also used TargetScan software to identify the targeting relationship between miRNAs and downstream up-regulated genes [26].

### Mouse model of MI

Male mice were used in this research. The mice were housed in a colony room at a controlled temperature (22°C) and humidity, under a 12-h light/dark cycle, and with food and water freely available. All surgical procedures were carried out in accordance with the National Institutes of Health Guide for the Care and Use of Laboratory Animals. In brief, the mice were anesthetized by 3% pentobarbital sodium and then ligated the anterior descending branch of the left coronary artery (LAD) for 48 h to establish the in *vivo* MI models [27]. The sham group mice were opened the chest bur not ligated with LAD. The mice were randomly divided into three groups as follows: (1) the sham group, which underwent sham operation and received vehicle (PBS, caudal vein injection); (2) the model group, which was subjected to LAD and received vehicle (PBS, caudal vein injection); and (3) the siRNA group, which were subjected to LAD and treated with siRNA of lncRNA *Gm47283* (30 nM siRNA dose per mice every day for one week, caudal vein injection).

### Measurement of infarct size

The heart was stained with TTC (triphenyltetrazolium chloride, Sigma-Aldrich, USA), and the size of the infarct was measured. After the remaining blood was washed away, the heart was cut into 2-mm thick sections below the ligature and stained with 1% TTC at 37°C for 15 min. Infarct area is stainless while the non-infarct area is stained red. The left ventricle was separated, and the ischemic area of the ventricle was dissected. The infarct area was measured by the weight ratio of infarct area and left ventricle.

### HE and Masson staining

Frozen sections of heart tissue were performed according to Dang’s method [28]. The mice hearts were rapidly harvested, washed, and stored in 4% paraformaldehyde for 48 h. Then the heart was cross-cut into 2 mm thick sections for hematoxylin and eosin (HE) staining and Masson staining via staining kit (HE: G1120, Masson: G1340, Solarbio, Beijing, China). Stained sections were photographed by the light microscope (Hitachi, Tokyo, Japan) at 100x magnification.

### *Cell culture and in* vitro *cell models*

The HL-1 cell lines were cultured in 25 cm^2^ cell culture flask (Corning, NYC, USA) with Dulbecco’s Modified Eagle Medium (DMEM) (Invitrogen, Waltham, USA) containing 15% fetal bovine serum (Invitrogen, Waltham, USA) at 37°C, 5% CO_2_ environment. In *vitro* MI models were established by H_2_O_2_ stimulation or hypoxia for 48 h.

### Vector construction and transfection

The full-length of lncRNA *Gm47283* was synthesized by PCR and inserted into pcDNA3.1 vector. Besides, pcDNA3.1 empty vector was used as a negative control. HL-1 was washed with serum-free medium and then incubated in 5 ml serum-free medium for 4–6 h for transfection. lncRNA *Gm47283* or control vectors (1 µg/mL) and x-treme GENE siRNA (Invitrogen, Carlsbad, USA) were separately mixed with 300 μl serum-free medium for 5 min. Then, two mixtures were combined and incubated at room temperature for 18 min. Finally, the plasmid mixture and x-treme GENE siRNA were added to the HL-1 and incubated at 37°C for 24 h [29].

### MiRNA mimic, siRNA construction and transfection

*MiR-706* mimics and small interfering RNA sequences that target to lncRNA *Gm47283* were synthesized by Gene Pharma (Gene Pharma, Shanghai, China). HL-1 was washed with serum-free medium and then incubated in 5 mL serum-free medium for 4–6 h for transfection. *MiR-706* mimics or control vectors (1 µg/mL) and x-treme GENE siRNA (Invitrogen, Carlsbad, USA) were separately mixed with 300 μl serum-free medium for 5 min. Then, two mixtures were combined and incubated at room temperature for 18 min. Finally, the mixture and x-treme GENE siRNA were added to the HL-1 and incubated at 37°C for 24 h.

### Luciferase reporter assays

The *Ptgs2ʹ*UTR and lncRNA *Gm47283* full-length sequence containing *miR-706* binding sites were amplified by PCR. For luciferase assay, cells were seeded in 48-well plates in triplicate, and 40 ng/well luciferase reporter vector, 10 pmol *miR-706* mimic or mimic control were co-transfected into plates using lipofectamine 2000 (Invitrogen, Carlsbad, USA). Cells were lysed at 24 h after transfection, and luciferase activity was determined using the Dual Luciferase Reporter Assay kit (Promega, Madison, USA).

### Reactive oxygen species (ROS) production assay

HL-1 cell lines were removed the medium and incubated with diluted DCFH-DA (S0033M, Beyotime, Shanghai, China) at 37°C for 20 min. After incubation, the cell lines were washed again. Fluorescence intensity of intracellular ROS was detected by fluorescence microscope (Leica, Heidelberg, Germany) at excitation wavelength of 488 nm.

### Measurement of the MDA and SOD levels

Malondialdehyde (MDA) and superoxide dismutase (SOD) were considered as the oxidative stress injury markers. Therefore, the quantities of MDA and SOD were measured by MDA and SOD kits (MDA: S0131S, SOD: S0101S, Beyotime, Shanghai, China) based on the manufacturer’s instructions.

### Western blot

Total protein was extracted from the left ventricular myocardium or HL-1 cell lines and lysed via RIPA buffer. Degenerated protein concentration was measured by bichinchoninic acid (BCA) Protein Assay Kit (Beyotime, Shanghai, China). In each experiment, 20 μg proteins were separated by sodium dodecyl sulfate-polyacrylamide gel electrophoresis (SDS-PAGE, 12% or 10% polyacrylamide gels) and transferred to nitrocellulose filter membrane. The membrane was blocked for 2 h with 5% skim milk in PBS at room temperature and then incubated at 4°C overnight with primary antibodies *Gpx4* (1:500 dilution, A11243, Abclonal, Wuhan, China), *Alox15* (1:1000 dilution, A6864, Abclonal, Wuhan, China), *GAPDH* (1:2000 dilution, A19056, Abclonal, Wuhan, China), and *Ptgs2* (1:1000 dilution, A1253, Abclonal, Wuhan, China). HRP Goat Anti-Rabbit IgG (H + L) antibody (1:5000 dilution, AS014, Abclonal, Wuhan, China) was used as the secondary antibody for 1 h at room temperature. The images were captured by Odyssey v3.0 software. Protein bands were measured by the Image J software.

### Quantitative real-time RT-PCR

Total RNA was extracted from the left ventricles and HL-1 cell lines using Trizol reagent (Invitrogen, Waltham, USA) according to manufacturer’s protocols. cDNA was synthesized by reverse transcription reagent kit (TAKARA, RR037A, Shiga, JAPAN). Gene expression was quantified by SYBR Green PCR Master Mix, and detected using ABI 7300 systems. U6 or GAPDH was served as an internal control for miRNAs and lncRNAs/mRNAs, respectively. 2^−ΔΔCt^ relative quantification method was used to calculate gene expression. Primer sequences are listed in Table S3.

### Preparation of stem-cell-membrane vesicles

Stem-cell membranes isolated from bone-marrow-derived mesenchymal stem cells according to the published method [30]. When the cells achieved 80% confluence, the stem cells were detached, centrifuged, and lysed. Then, the stem cell membrane was collected by centrifuging at 15,000 g for 30 min. To prepare stem-cell-membrane vesicles (SVs), the obtained stem-cell membranes were dispersed in siRNAs and extruded using a physical extrusion method.

To prepare DiR-labeled SVs, Briefly, a post-insertion technique was used. DiR dye was dissolved in ethanol (5 mg/mL). A total of 100 μL of the DiR solution was then gradually added to 1 mL of the SVs suspensions and incubated at 37°C to obtain DiR-labeled SVs. The ethanol was then removed by rotary evaporation.

### Statistical analysis

Data were presented as the Mean ± SEM and analyzed by GraphPad Prism 8.0 software. One-way ANOVA method was used to compare between experimental groups. *P* < 0.05 was considered as statistically significant.

### Results

Previous studies have found that lncRNAs play crucial roles in the regulatory processes of multiple cardiovascular diseases. Here, we performed a bioinformatics analysis to infer the potential pathogenic lncRNAs and found lncRNA Gm47283 was the risk factor in MI. Furthermore, we also found some crucial genes were also dysregulated in MI. We hypothesized that lncRNA might participate in the regulatory processes by regulating these crucial genes. Thus, we performed the molecular biology experiments to reveal the molecular mechanism of lncRNA Gm47283 in MI.

### Identification of differentially expressed transcripts in MI

**Figure 1.**
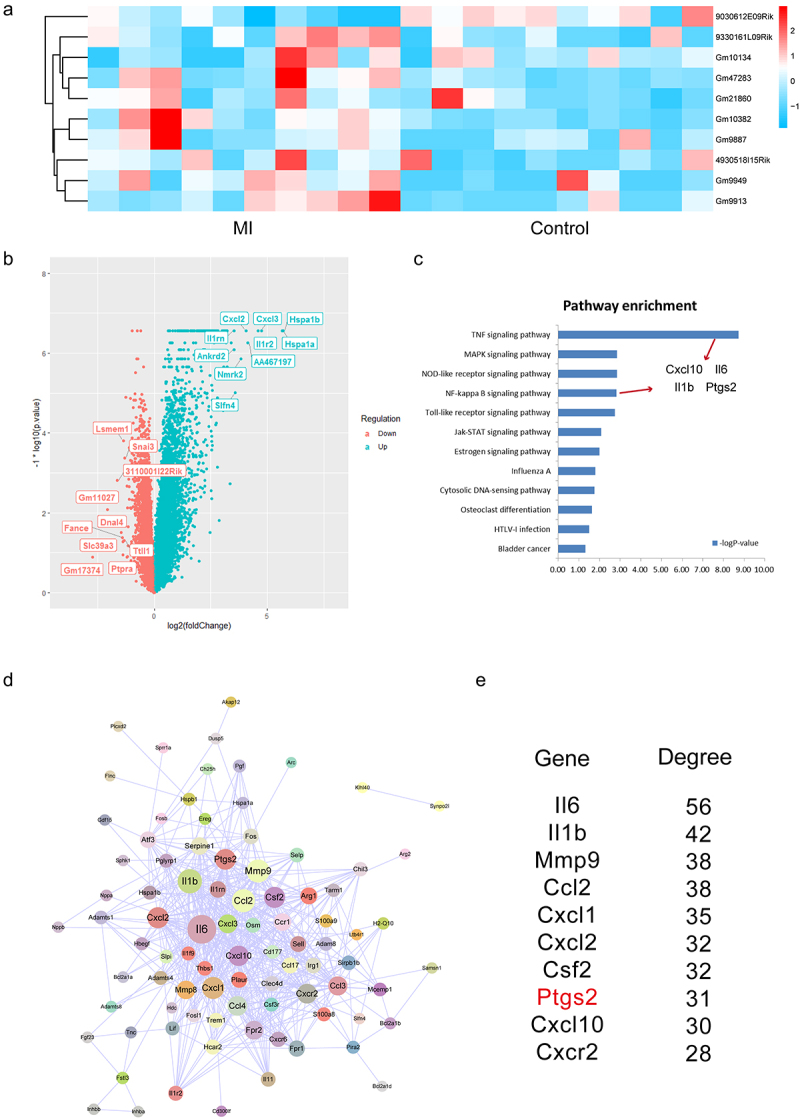
**Identification of differentially expressed genes in MI**. (a) The heatmap of top 10 differentially expressed lncRNAs in MI from 20 samples. (b) The volcano plot of differentially expressed genes. Top 10 up-regulated genes and down-regulated genes were labeled in the plot. (c) Pathway enrichment analysis of up-regulated genes. Four common genes between *TNF* signaling and *NF-κB* signaling pathways were labeled. (d) The visualization of up-regulated genes-induced PPI network. (e) Degree analysis of the PPI network.

Firstly, we used the bioinformatics pipeline to identify differently expressed genes and lncRNAs at the threshold of |Fold change| >1.5 or *P*-value <0.05. Here, because of the annotation rates for the dataset, the down-regulated lncRNAs were not identified at the strict thresholds. Therefore, we focused on mining the regulatory information for the dysregulated lncRNAs and genes. As a result, 10 lncRNAs were extracted and considered as the risk factors in MI, including 9 up-regulated lncRNAs and 1 down-regulated lncRNA (Figure 1a). We also identified 659 up-regulated genes, which were significantly enriched in the pathway of *TNF* signaling, *MAPK* signaling, and *NF-κB* signaling pathway (Figure 1b and 1c) [31–34]. Interestingly, we then extracted the enriched genes in each significant pathways, and results showed that four genes with known function were identified, including *Cxcl10, Il6, Il1β,* and *Ptgs2* (Figure 1c). In addition, to systematically investigate the up-regulated genes in MI, we input all these up-regulated genes into String database and extracted the protein–protein interaction networks (Figure 1d). In the network, we found that some genes were occupied high node degrees, indicating the crucial functions of which they had.

Interestingly, we then used miRanda software to predict the relationship between lncRNAs and miRNAs and used targetScan software to predict the relationship between miRNAs and 4 known genes in Figure 1c. As a result, we found that *miR-706* is the potential mediator between lncRNA *Gm47283* and *Ptgs2* (Supplementary Table S1 and S2).

### *LncRNA* Gm47283 *and* Ptgs2 *were up-regulated in MI*

**Figure 2.**
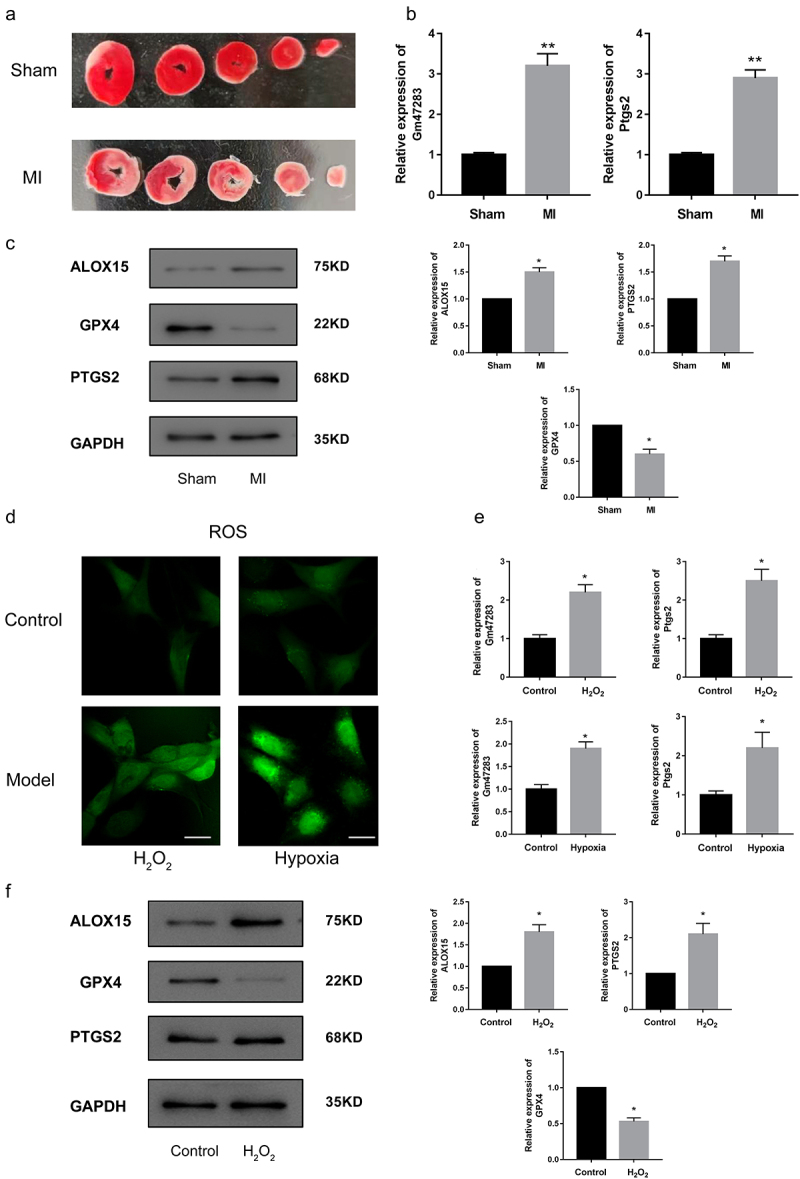
**Validation of the expression of lncRNAs and downstream genes in MI**. (a) TTC staining of sham hearts and model hearts. (b) Real-time PCR results of lncRNA *Gm47283* and *Ptgs2*. **P < 0.01 vs Sham group. n = 6. (c) Western blot results of *Ptgs2, Alox15* and *Gpx4* expression. n = 6. (d) ROS staining results of control cardiomyocytes and cardiomyocytes induced by H_2_O_2_ and hypoxia. n = 6. (e) Real-time PCR results of lncRNA *Gm47283* and *Ptgs2* in *vitro* groups. *P < 0.05 vs Control group. n = 6. (f) Western blot results of *Ptgs2, Alox15* and *Gpx4* expression. n = 6. Scale bars; 25 μm.

Recently, the ceRNA mechanism of lncRNAs has attracted a lot of research interest. The concept of ceRNAs demonstrates that the RNA molecules containing the binding sites (sequence complementarity) to a particular miRNA can competitively bind to this individual miRNA to reduce its functional availability [35]. LncRNAs can often act as ceRNAs, sponging and sequestering miRNAs, indirectly regulating the target genes of miRNAs, or specifically, releasing the target genes from repression by the targeted miRNA[36]. Next, we prepared the MI models to investigate the regulatory role of lncRNA *Gm47283* and *Ptgs2* in *vivo* and *vitro*. TTC staining showed that significant infarct areas in model groups (Figure 2a), indicating our in *vivo* models were constructed successfully. Then we used Real-time PCR to detect the expression of lncRNA *Gm47283* and *Ptgs2* in models, and results showed that the two risk genes were both up-regulated in MI (Figure 2b). Western blots results also validated that *Ptgs2* was up-regulated in models (Figure 2c and Supplementary Figure S1). In addition, we also detected the expression of *Alox15* and *Gpx4* (Figure 2c and Supplementary Figure S1). Results showed that *Alox15* was up-regulated in MI. *Gpx4* was repressed in MI, indicating that ferroptosis activity was activated in MI.

Second, we also used H_2_O_2_ and hypoxia to induce in *vitro* myocardial damage models to detect the expression of lncRNA *Gm47283* and *Ptgs2*. ROS kit results showed that ROS was activated by treating H_2_O_2_ and hypoxia (Figure 2d). LncRNA *Gm47283* and *Ptgs2* were also up-regulated in cellular hypoxia (Figure 2e). Moreover, western blot results showed that *Ptgs2* and *Alox15* were increased in models but *Gpx4* was decreased (figure 2f and Supplementary Figure S1), which was in accordant with in *vivo* model results. We also detected the activity of SOD and MDA. Results showed that SOD was decreased and MDA was increased after H_2_O_2_ treatment. Above results validated the predictive factors were up-regulated and ferroptosis was also activated in MI.

### *LncRNA* Gm47283 *participated in the pathological processes of MI*

**Figure 3.**
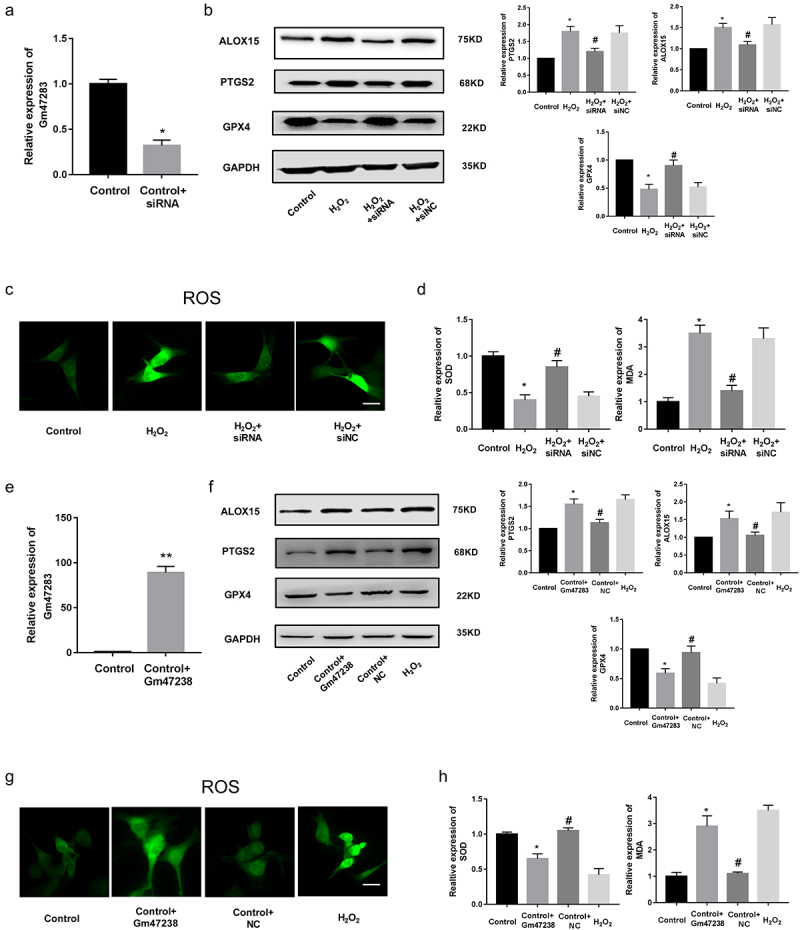
**Knockdown of lncRNA *Gm47283* alleviates MI**. (a) Real-time PCR results of lncRNA *Gm47283* knockdown. *P < 0.05 vs Control group. n = 6. (b) Western blot results of *Ptgs2, Alox15* and *Gpx4* expression in lncRNA knockdown groups. n = 6. (c) ROS staining results of cardiomyocytes in knockdown groups. n = 6. (d) Detection of SOD and MDA levels in lncRNA knockdown groups. *P < 0.05 vs Control group, ^#^*P* < 0.05 vs H_2_O_2_ group. n = 6. (e) Real-time PCR results of lncRNA *Gm47283* overexpression. **P < 0.01 vs Control group. n = 6. (f) Western blot results of *Ptgs2, Alox15* and *Gpx4* expression in lncRNA overexpression groups. n = 6 (g) ROS staining results of cardiomyocytes in lncRNA overexpression groups. n = 6. (h) Detection of SOD and MDA levels in lncRNA overexpression groups. * *P* < 0.05 vs Control group, ^#^*P* < 0.05 vs Control+*Gm47283* group. n = 6. Scale bars; 25 μm.

The role of lncRNA *Gm47283* in MI is unclear. We then investigated the function of lncRNA *Gm47283* via loss-of-function and gain-of-function experiments in *vitro*. First, we constructed a siRNA to inhibit the expression of lncRNA *Gm47283*. Real-time PCR results validated that lncRNA *Gm47283* was efficiently repressed (Figure 3a). Western blot results showed that *Ptgs2* was decreased with lncRNA knockdown. Ferroptosis marker *Alox15* was up-regulated and *Gpx4* was down-regulated (Figure 3b and Supplementary Figure S1). Furthermore, ROS staining results showed that inhibition of lncRNA *Gm47283* could inhibit the ROS activity (Figure 3c). LncRNA *Gm47283* knockdown could also increase the quantity of SOD and decrease the quantity of MDA (Figure 3d). These results indicated that inhibition of lncRNA *Gm47283* could attenuate cardiomyocyte cellular hypoxiavia repressing ferroptosis. We also overexpressed lncRNA *Gm47283* expression via construction of plasmid and Realtime PCR results validated that lncRNA was overexpressed in cardiomyocytes (Figure 3e). We found that over-expression of lncRNA *Gm47283* significantly increased the expression of *Ptgs2* and *Alox15* and repressed the expression of *Gpx4* (figure 3f and Supplementary Figure S1). In addition, overexpression of lncRNA *Gm47283* could increase the level of ROS and MDA and decrease the level of SOD (Figure 3g and 3h). Above results indicated that lncRNA *Gm47283* played an impaired role in MI via regulating the ferroptosis of cardiomyocyte.

### MiR-706 *was the regulator of MI*

**Figure 4.**
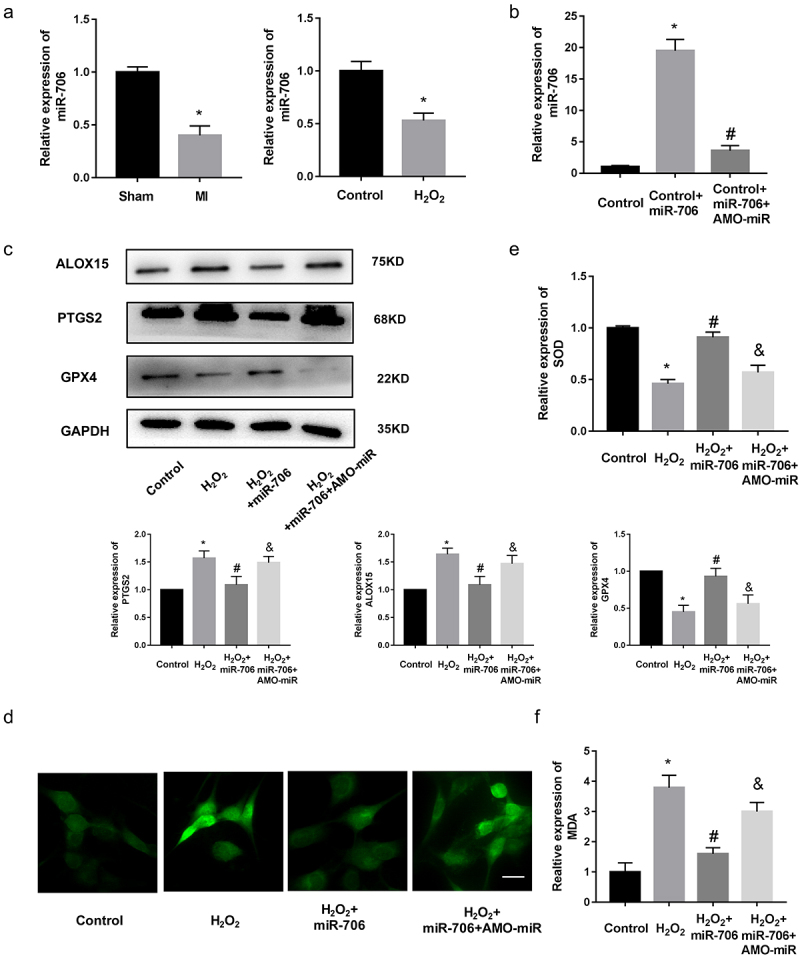
***miR-706* is the regulator of MI**. (a) Real-time PCR results of *miR-706* expression in MI models. *P < 0.05 vs Control/Sham group. n = 6. (b) Real-time PCR results of *miR-706* expression in miRNA interference groups. *P < 0.05 vs Control group. ^#^*P* < 0.05 vs Control+*miR-706* group. n = 6. (c) Western blot results of *Ptgs2, Alox15* and *Gpx4* expression in *miR-706* overexpression or inhibition groups. n = 6. (d) ROS staining results of cardiomyocytes in *miR-706* overexpression or inhibition groups. n = 6. (e-f) Detection of SOD and MDA levels in *miR-706* overexpression or inhibition groups. *P < 0.05 vs Control group, ^#^*P* < 0.05 vs H_2_O_2_ group, ^&^*P* < 0.05 vs H_2_O_2_+ *miR-706* group. n = 6. Scale bars; 25 μm.

microRNAs negatively regulate gene expression by promoting mRNA degradation or inhibiting mRNA translation [37]. Previous studies also have found that lncRNAs exerted functions by endogenous sponging miRNAs to influence the expression of downstream protein coding genes. Thus, we speculated that lncRNA *Gm47283* might regulate MI via ceRNA mechanism. Based on our prediction, we considered that *miR-706* was the downstream target of lncRNA *Gm47283. MiR-706* was demonstrated as the regulator of oxidative stress in liver fibrogenesis [38]. Then, we performed real-time PCR to detect the expression of *miR-706* in MI models. Results showed that *miR-706* was down-regulated in MI models (Figure 4a). To investigate the regulatory role of *miR-706* in MI, we constructed the *miR-706* mimics and AMO-*miR-706* to overexpress and inhibit the expression of *miR-706*. Real-time PCR results showed that *miR-706* was induced by mimics and repressed by AMO-miR (Figure 4b). Western blot results showed that *miR-706* could inhibit the expression of *Ptgs2* and AMO-miR could reverse the repression effects. Ferroptosis marker *Alox15* was also repressed by *miR-706. Gpx4* expression was increased after mimic treatment (Figure 4c and Supplementary Figure S1). We also detected the quantity of ROS after miRNA treatment. As a result, ROS staining results showed that overexpression of *miR-706* could decrease the ROS activity (Figure 4d). Overexpression of *miR-706* could also repress the quantity of MDA and increase the quantity of SOD (Figure 4e). These results indicated that *miR-706* was a protective regulator of MI, which participated in MI via regulating cellular ferroptosis level. Moreover, this result also implied that our hypothesis of lncRNA *Gm47283*/*miR-706*/*Ptgs2* regulatory axis in MI existed.

**Figure 5.**
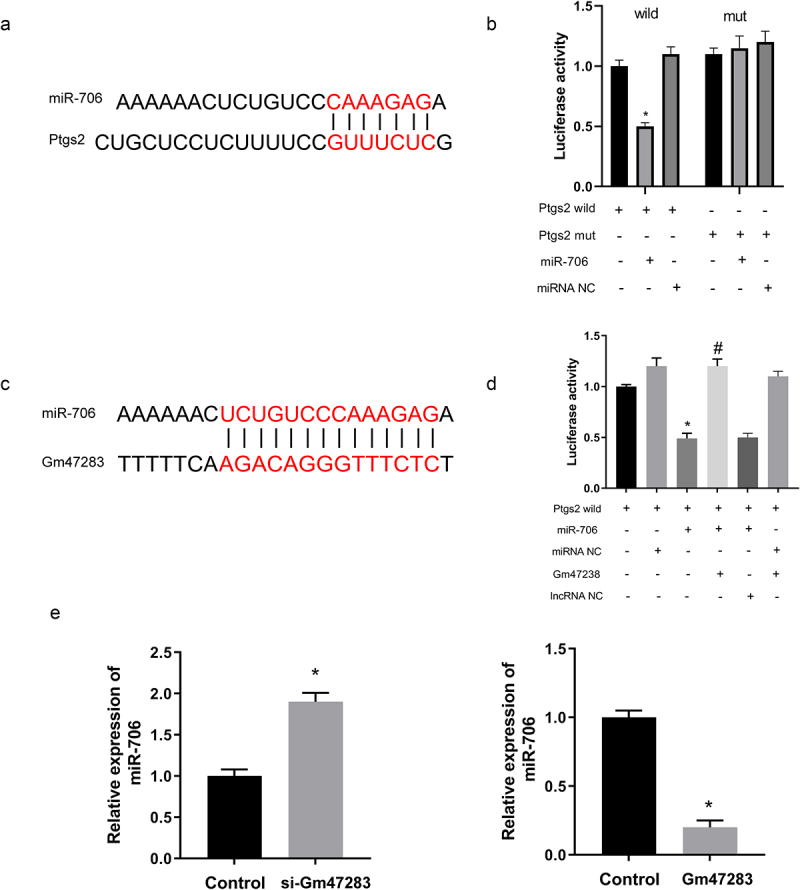
**Validation of the direct regulatory relationships among lncRNA, miRNA and mRNA**. (a) The interaction of *miR-706* and *Ptgs2*. (b) Validation of direct interactions between *miR-706* and *Ptgs2*. *P < 0.05 vs *Ptgs2*-wt group. n = 3. (c) The interaction of *miR-706* and lncRNA *Gm47283*. (d) Validation of direct interactions among lncRNA *Gm47283, miR-706* and *Ptgs2*. *P < 0.05 vs *Ptgs2*-wt group, ^#^*P* < 0.05 vs *Ptgs2*-wt+*miR-706*+ *Gm47283* group. n = 3. (e) Real-time PCR results of *miR-706* expression in lncRNA overexpression and knockdown groups. *P < 0.05 vs Control group. n = 6.

Next, we used luciferase report system to validate the relationship among lncRNA, miRNA, and downstream gene. Briefly, Figure 5a validates the direct binding relationship between *miR-706* and *Ptgs2*. Moreover, we inserted wild-type and mutated-type *Ptgs2* into luciferase plasmids. After co-transfection of *Ptgs2* wild/mut luciferase plasmids and *miR-706* mimic/miRNA NC into cells, results showed that *miR-706* mimic sharply repressed the luciferase activity of wide-type *Ptgs2* plasmid, but the activity of mutated-type *Ptgs2* was not changed with *miR-706* mimics (Figure 5b). Then, we established lncRNA *Gm47283* plasmid and transfected the plasmid into the luciferase system (Figure 5c). Results showed that lncRNA *Gm47283* could rescue the degradation of *miR-706* to *Ptgs2* (Figure 5d). Furthermore, we also detected the expression of *miR-706* expression was regulated after lncRNA *Gm47283* inhibition or overexpression (Figure 5e).

### *LncRNA* Gm47283 *regulates MI via* miR-706 *and* Ptgs2

**Figure 6.**
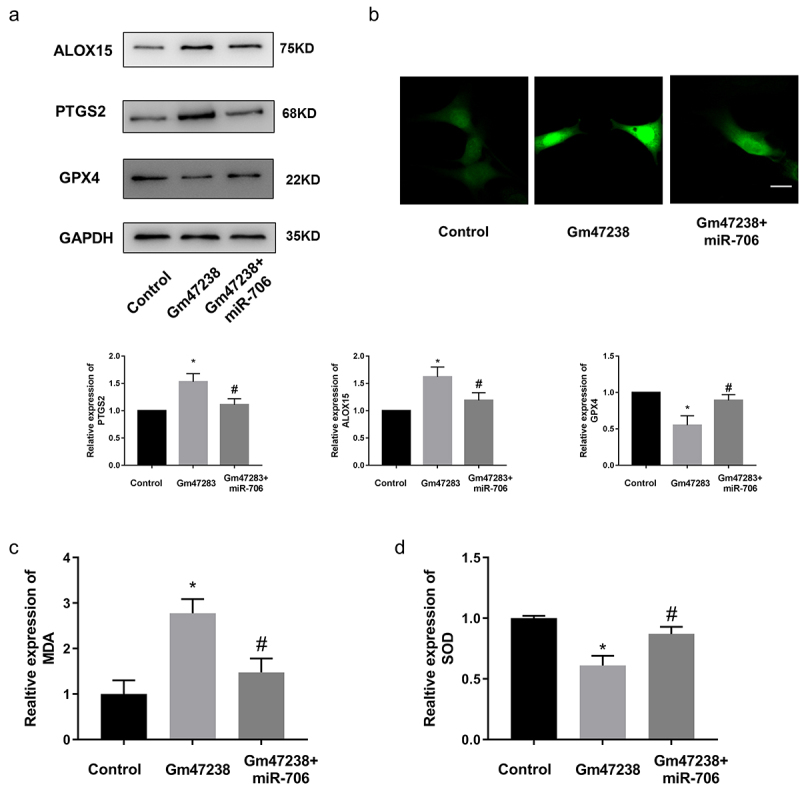
**LncRNA *Gm47283* regulates MI via *miR-706* and *Ptgs2***. (a) Western blot results of *Ptgs2, Alox15* and *Gpx4* expression in co-transfection group. n = 6. (b) ROS staining results of cardiomyocytes in co-transfection groups. n = 6. (c-d) Detection of SOD and MDA levels in co-transfection groups. *P < 0.05 vs Control group, ^#^*P* < 0.05 vs *Gm47283* group. n = 6. Scale bars; 25 μm.

To further investigate the regulatory axis of lncRNA *Gm47283*/*miR-706*/*Ptgs2* in MI, we performed a co-transfection experiment via co-transfecting lncRNA *Gm47283* and *miR-706* into cardiomyocyte. Results showed that the enhanced expression of *Ptgs2* by lncRNA *Gm47283* overexpression was repressed by *miR-706* supplement (Figure 6a and Supplementary Figure S1). We also detected the expression of ferroptosis marker expression via western blot. As a result, we found that *Alox15* expression was elevated by lncRNA *Gm47283* and then was repressed by *miR-706. Gpx4* expression was inhibited by lncRNA overexpression and was rescued by *miR-706* (Figure 6a and Supplementary Figure S1). Additionally, we also detected the quantities of ROS, MDA, and SOD. Results showed that *miR-706* could suppress the lncRNA *Gm47283* mediated increases of ROS and MDA (Figure 6b-6c). *MiR-706* could also rescue the decreased SOD level that was decreased after lncRNA overexpression (Figure 6d). These results indicated that lncRNA *Gm47283* could regulate MI via *miR-706*/*Ptgs2*/ferroptosis axis.

### *Inhibition of lncRNA* Gm47283 *attenuated MI in* vivo

**Figure 7.**
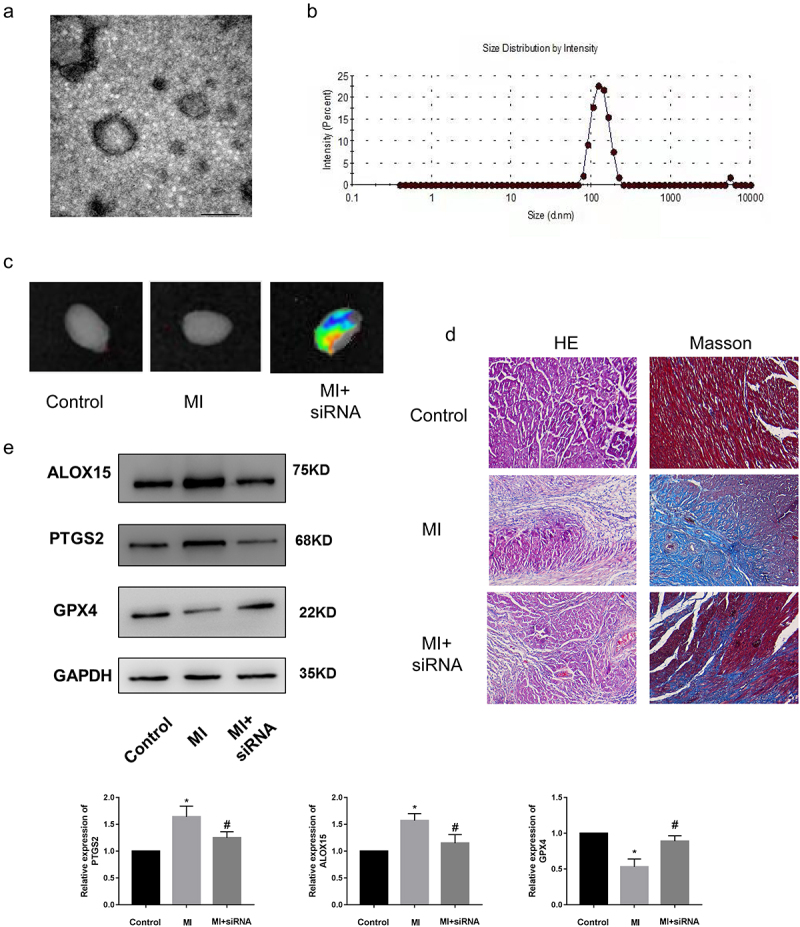
**Knockdown of lncRNA *Gm47283* via stem cell membrane coated siRNA attenuates MI in *vivo***. (a) Electron microscopy characterization of the stem cell membrane coated siRNA. (b) Size distribution of the stem cell membrane coated siRNA. (c) In *vivo* imaging of the hearts in different groups. (d) Masson and HE staining of hearts in different groups. n = 6. Western blot results of *Ptgs2, Alox15* and *Gpx4* expression in *vivo* knockdown group. n = 6.

Currently, the regulatory mechanisms of a series of functional ncRNAs have been widely elucidated, but their clinical application has been greatly hindered due to little attention paid to the development of effective in *vivo* delivery strategies to regulate the biological functions. Moreover, compared to the billions of intracellular ncRNAs that may exert their biological functions, the proportion of ncRNAs currently found to be related to MI is still very low. Therefore, the regulatory mechanism and effective delivery of more functional ncRNAs remain to be explored. We have demonstrated that lncRNA *Gm47283* regulated MI via *miR-706*/*Ptgs2*/ferroptosis axis. We then performed in *vivo* inhibition experiments to validate the therapeutic effects of lncRNA *Gm47283* in MI models. Here, we used stem cell membrane coated siRNA to target the injury regions. Material characterization experiments showed that the stem cell membrane was successfully coated the siRNA (Figure 7a-7b). More importantly, we also demonstrated that the coated siRNA was aggregated in heart (Figure 7c). After 1 weeks modeling, we found that stem cell coated siRNA could repress the inflammatory infiltration and collagen formation based on HE Staining and Masson staining (Figure 7d). Furthermore, we also detected the expression of *Ptgs2* and ferroptosis marker proteins (*Alox15* and *Gpx4*). Results showed that inhibition of lncRNA *Gm47283* could decrease the expression of *Ptgs2* and *Alox15* and rescue the expression of *Gpx4* (Figure 7e and Supplementary Figure S1). These results suggested that heart targeted lncRNA *Gm47283* inhibition could be used an effective drug to attenuate MI.

## Discussion

**Figure 8.**
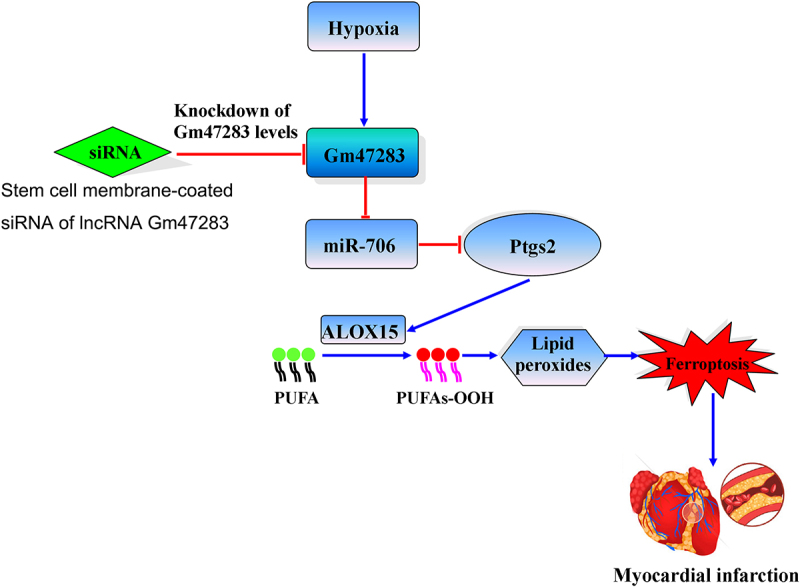
The mimic mechanism of lncRNA *Gm47283*/*miR-706*/*Ptgs2*/ferroptosis axis in MI.

MI is the ischemic necrosis of myocardium, on the basis of coronary artery pathological changes, the blood flow of coronary artery is sharply reduced or interrupted, so that the corresponding myocardium appears serious and permanent acute ischemia, eventually bring about the ischemic necrosis of myocardium. It is one of the leading causes of death. In this study, we performed bioinformatics analysis to identify risk lncRNAs in MI and investigated the biological function and mechanism of lncRNA *Gm47283* in rat models in *vivo* and in *vitro*. As a result, 10 lncRNAs were identified as risk factors from array data. Additionally, we found that *TNF* and *NF-κB* pathways were activated in MI, four protein coding genes of which were also extracted, including ferroptosis gene *Ptgs2*. In *vivo* and in *vitro* models also demonstrated that lncRNA *Gm47283* and *Ptgs2* were activated in MI. Interference of lncRNA *Gm47283* expression has the impact on *Ptgs2* expression and ferroptosis activity. Mechanically, we found that lncRNA *Gm47283* participated in MI through regulating cardiomyocyte ferroptosis activity by targeting *miR-706*, which could target to the 3ʹUTR of *Ptgs2*. Moreover, inhibition of lncRNA *Gm47283* by stem cell membrane coated siRNA could attenuate MI in *vivo* (Figure 8).

Recent studies on miRNAs have renewed our understanding about the regulation of MI. Various studies have provided strong evidence that miRNAs play important roles in MI [39,40]. Notably, the expression of *miR-706* was significantly down-regulated in the mice model of MI. In addition, *miR-706* in cardiomyocytes was resistant to H_2_O_2_-induced damage. Furthermore, more importantly, we found that forced expression of *miR-706* attenuated cardiomyocyte ferroptosis. Our study elucidated the anti-ferroptotic effect and mechanisms of *miR-706* in MI, and indicated that the exogenous application of *miR-706* might be a novel therapeutic strategy for MI. In the present study, enforced expression of *miR-706* resulted in a reduction of *Ptgs2* in cardiomyocytes. We also verified the interaction of *Ptgs2* and *miR-706* by luciferase assay. This indicated that the interaction between *miR-706* and *Ptgs2* 3ʹUTR actually existed *in vitro*. Furthermore, we also investigate the effects of *miR-706* negative control on *Ptgs2*, results showed that the negative control could not affect the expression of *Ptgs2* and decrease ROS accumulation (Supplementary Figure S2).

LncRNAs were considered as the crucial regulators in gene post-transcription regulation. In the field of MI, numerous of lncRNAs were demonstrated to participate in the disease regulatory processes via multiple pathways, such as inflammation, apoptosis, and autophagy. Thus, in this study, we wanted to find a novel lncRNA transcript of MI and unveil the regulatory mechanism of it. We firstly performed bioinformatics analysis to call the differentially expressed lncRNAs. Ten up-regulated lncRNAs were identified as risk factors. Real-time PCR results showed that lncRNA *Gm47283* was up-regulated in myocardial models in *vivo* and in *vitro*. But the regulatory mechanism of lncRNA *Gm47283* is unknown. Interestingly, we found that numerous mRNAs were also up-regulated in MI, which were enriched in *TNF* signaling pathway, *MAPK* signaling pathway, and *NF-κB* signaling pathways. Moreover, we found that four up-regulated genes were both enriched in *TNF* and *NF-κB* signaling pathways and showed a strong connective activity in the differentially expressed genes-induced PPI network. Whether lncRNA *Gm47283* regulated MI via these genes attracted our attention. Based on comprehensive bioinformatics analysis, we found that lncRNA *Gm47283* could target to *miR-706* to interfere the expression of *Ptgs2*, which was a known marker of cellular ferroptosis. Thus, we speculated that lncRNA *Gm47283* involved in MI via *miR-706*/*Ptgs2*/ferroptosis axes.

Ferroptosis is a novel form of regulated cell death identified as iron-dependence, lipid peroxidation collection, and reactive oxygen species (ROS) generation. It is remarkably distinct from other types of classical regulated cell death, such as apoptosis, necrosis, and necroptosis. Ferroptosis participated in various diseases, including cancer, Parkinson’s disease, stroke, and IRI. However, its effect in the progression of AMI remains unclear. Here, we found that *Ptgs2* was up-regulated in MI and *Ptgs2* was predicted as the downstream target of lncRNA *Gm47283*/*miR-706* axis. *Ptgs2* is also known as *COX2*, which is a well-accepted biomarker for ferroptosis onset. *COX2* is induced in cells undergoing ferroptosis [41]. *Alox15* was demonstrated as the downstream proteins of *COX2* [42,43]. Results showed that *Ptgs2* and *Alox15* were both up-regulated in MI. In *vitro* loss and gain function experiments also validated that *Ptgs2* participated in MI via serving as the downstream gene of lncRNA *Gm47283*.

At present, many functional lncRNAs are known, and their regulatory mechanisms have been widely elucidated. However, few efforts have been made to develop effective *in vivo* regulatory strategies to improve treatment outcomes. RNA interference is the most convenient method because of its strong ability to silence target gene expression. However, due to the polyanion and biomacromolecule characteristics of RNA interference therapies such as siRNA, specific delivery vehicles are required to facilitate siRNA delivery *in vivo* [44,45]. Nanotechnology has shown great promise for improvement of *in vivo* siRNA delivery, and several RNA interference nanoparticle platforms have been marketed or entered into early phase clinical trials for the treatment of various diseases. However, effective and safe systemic delivery of siRNAs into heart *in vivo* remains challenging, due to the complexities of cardiovascular disease.

In this study, we used stem cell membrane coated siRNA to inhibit the expression of lncRNA *Gm47283*. Stem cell membrane targeted therapy is considered as an advanced tool to deliver the drugs to the injury regions. Thus, here we selected this method and results showed that the stem cell membrane coated siRNA was directly targeted to the injury heart.

## Conclusion

Taken together, we identified lncRNA *Gm47283* was the top risk factor in MI. Bioinformatics analysis predicted that lncRNA *Gm47283* exerted function via targeting *miR-706* and *Ptgs2. Ptgs2* was also the known regulator of ferroptosis. Inhibition or overexpression of lncRNA *Gm47283* could regulate *Ptgs2* expression and downstream ferroptosis activity. Overexpression of *miR-706* could inhibit the expression of *Ptgs2* and the activity of ferroptosis. Our study demonstrated a novel regulatory axis containing lncRNA *Gm47283*/*miR-706*/*Ptgs2*/ferroptosis in MI, which provided a potential mechanism for heart therapeutic treatment in clinic.

## Supplementary Material

Supplemental Material
