# Supplementary figures and images for: Suppression of lncRNA Gm47283 attenuates myocardial infarction via miR-706/ Ptgs2/ferroptosis axis
Source: Bioengineered. 2022 Apr 29;13(4):10786–802. doi: 10.1080/21655979.2022.2065743 (PMC9208485; doi:10.1080/21655979.2022.2065743)

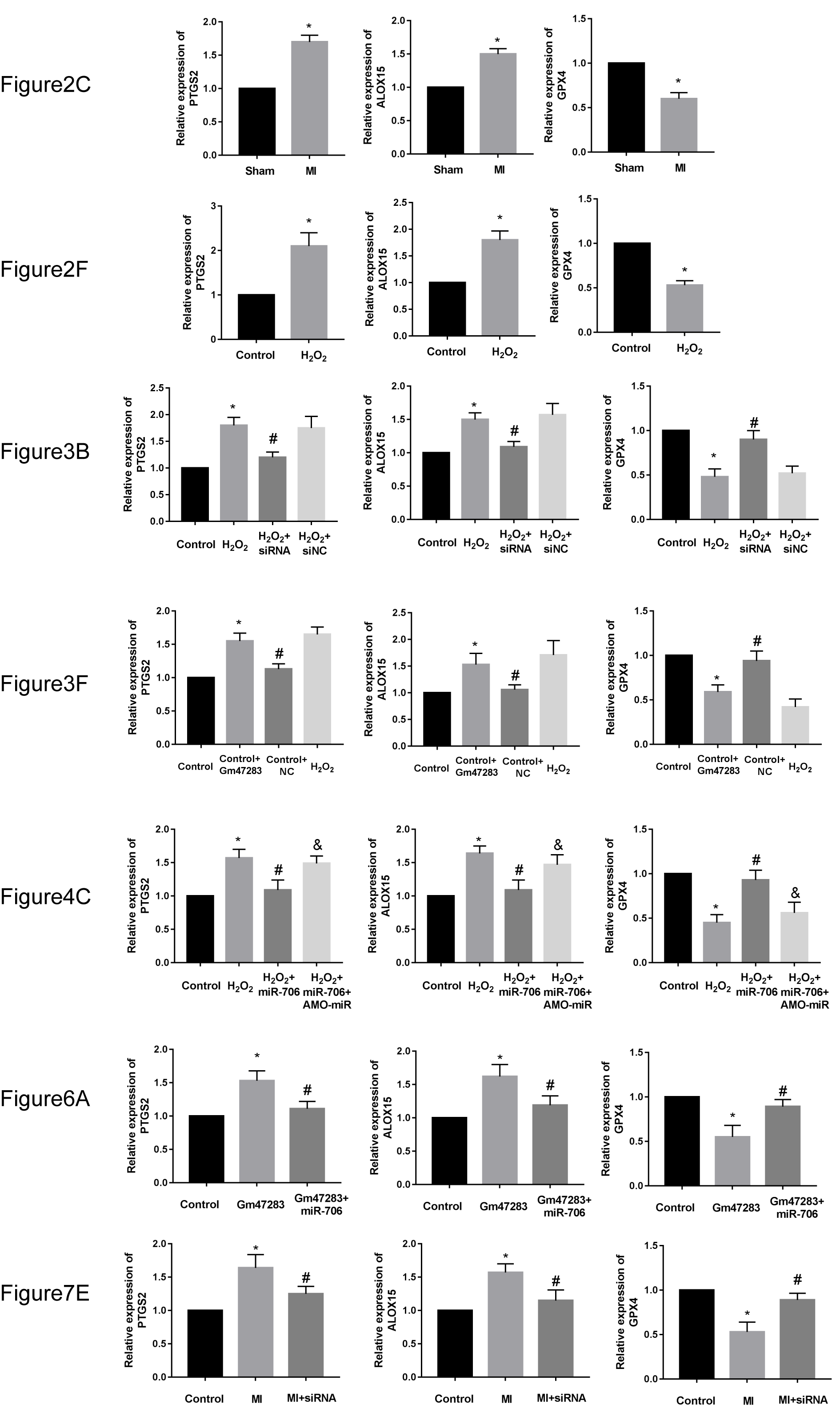

Supplement: Supplemental Material [file KBIE_A_2065743_SM1174.zip › supplementary/Supplementary Fgiure S1.tif]

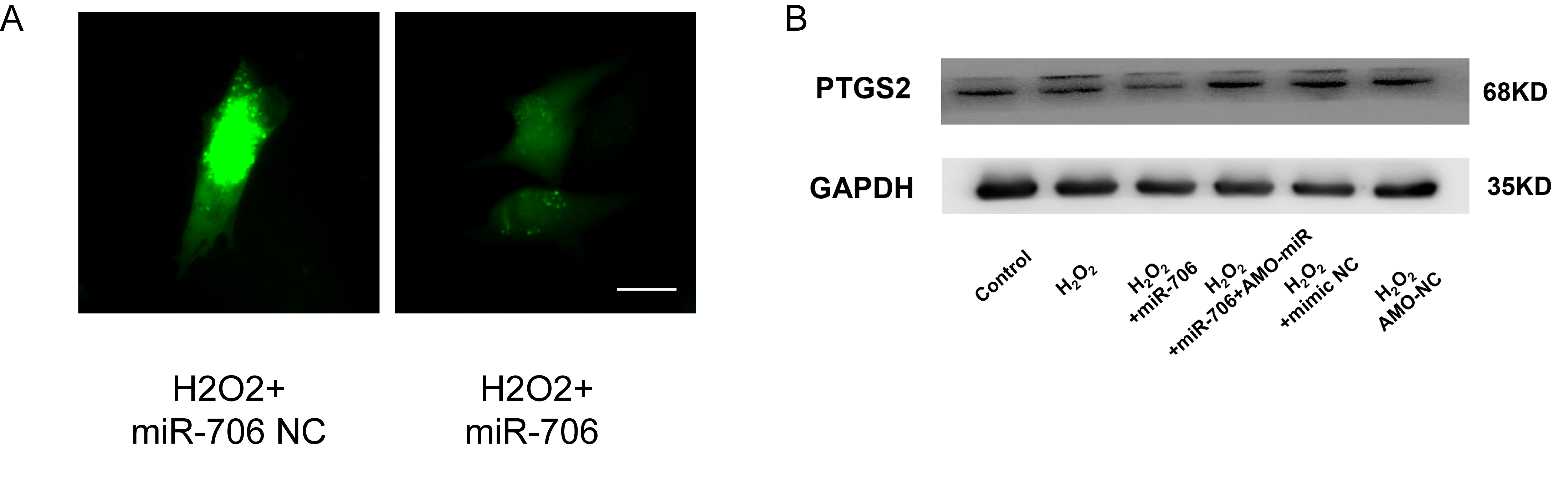

Supplement: Supplemental Material [file KBIE_A_2065743_SM1174.zip › supplementary/Supplementary Fgiure S2.tif]
